# Estimation of Psychological Distress in Japanese Youth Through Narrative Writing: Text-Based Stylometric and Sentiment Analyses

**DOI:** 10.2196/29500

**Published:** 2021-08-12

**Authors:** Masae Manabe, Kongmeng Liew, Shuntaro Yada, Shoko Wakamiya, Eiji Aramaki

**Affiliations:** 1 Nara Institute of Science and Technology Nara Japan

**Keywords:** psychological distress, youth, narratives, natural language processing, Japan, mental health, stress, distress, young adult, teenager, sentiment

## Abstract

**Background:**

Internalizing mental illnesses associated with psychological distress are often underdetected. Text-based detection using natural language processing (NLP) methods is increasingly being used to complement conventional detection efforts. However, these approaches often rely on self-disclosure through autobiographical narratives that may not always be possible, especially in the context of the collectivistic Japanese culture.

**Objective:**

We propose the use of narrative writing as an alternative resource for mental illness detection in youth. Accordingly, in this study, we investigated the textual characteristics of narratives written by youth with psychological distress; our research focuses on the detection of psychopathological tendencies in written imaginative narratives.

**Methods:**

Using NLP tools such as stylometric measures and lexicon-based sentiment analysis, we examined short narratives from 52 Japanese youth (mean age 19.8 years, SD 3.1) obtained through crowdsourcing. Participants wrote a short narrative introduction to an imagined story before completing a questionnaire to quantify their tendencies toward psychological distress. Based on this score, participants were categorized into higher distress and lower distress groups. The written narratives were then analyzed using NLP tools and examined for between-group differences. Although outside the scope of this study, we also carried out a supplementary analysis of narratives written by adults using the same procedure.

**Results:**

Youth demonstrating higher tendencies toward psychological distress used significantly more positive (happiness-related) words, revealing differences in valence of the narrative content. No other significant differences were observed between the high and low distress groups.

**Conclusions:**

Youth with tendencies toward mental illness were found to write more positive stories that contained more happiness-related terms. These results may potentially have widespread implications on psychological distress screening on online platforms, particularly in cultures such as Japan that are not accustomed to self-disclosure. Although the mechanisms that we propose in explaining our results are speculative, we believe that this interpretation paves the way for future research in online surveillance and detection efforts.

## Introduction

### Background

Adolescents often display premonitory symptoms of mental illnesses arising from psychological distress (such as depression and anxiety-related disorders) [1-3]. Noticing the onset of such symptoms is important to facilitate effective treatment and diagnosis. Any delay between the onset of psychopathological symptoms to diagnosis and clinical treatment can result in a worsening of the condition, leading to complications not only for the individual’s mental health but also with respect to their physical health and social relationships [4]. In particular, youth tend to report more symptoms of internalizing mental illnesses that are related to psychological distress [5] and accompanying physical or vegetative changes (eg, weight and appetite changes) compared to adults. Consequently, as with any psychopathology, early access to medical and psychological assistance is important for the recovery process. Accordingly, the development of methods for the early detection of psychological distress in youth is of crucial importance.

### Text-Based Screening

In recent years, the rapid development of natural language processing (NLP) technology has enabled screening for dementia or depression through examinations of language use in free-text tasks [6-8]. One proposal to increase the effectiveness of such examinations is to limit and control the topics of these free-text tasks. This would reduce the occurrence of situations where differences in language use are due to differences in writing tasks and topics, which may confound language use detection with psychopathology. For example, the written narrative in recollecting a painful experience may be very different from the narrative recounting an interesting episode heard from a friend. Regardless of the underlying mental health of both instances, it is highly likely that different language and terms would be used in the recounting of these memories.

In this study, we adopted NLP techniques for detecting psychological distress disorders in young Japanese writers, and then describe possible language indicators that may reflect these differences. Accordingly, we consider the characteristics of youth with mental illness, and propose that writing fictional, imaginative narratives may appropriately allow for the generation of content that facilitates the detection of psychological distress.

### Psychopathology and Autobiographical Narratives

Prior research on narrative writing and psychopathology detection has focused on autobiographical narratives stemming from self-disclosure. For example, when a patient with depression (ie, major depressive disorder) recalls their own experience, they tend to rely on general or repeated memory (eg, I played a game with friends last week) rather than concrete memory (eg, I played volleyball with Sato last Thursday) or extended memory (eg, looking at this souvenir reminded me of my cruise trip last winter). This phenomenon is referred to as overgeneral autobiographical memory [9-11] and has been well-documented even in youth with depression [12].

However, detection of psychopathology in autobiographical self-disclosure–based narratives may not be as effective in Japan, given that the Japanese are generally less accustomed to self-disclosure [13,14]. Furthermore, self-disclosure on online platforms comes with risks such as cyberbullying and social isolation [15]. Thus, an alternative method of detection is needed. Toward this end, we propose the use of fictional, imaginative writing that does not require any form of self-disclosure. As a first step, such research would need to examine which part of the narrative affords detection of psychological distress in the writer in detail. We consider that story creation relies on one’s past experiences and memory, but in a transformed, unrecognizable manner, resembling a generalized experience that can be shared with others.

### Imaginative Narratives as Writing Topics

We propose that psychological distress in the writer may be observable through their written narratives. In other words, topic questions should encourage respondents to engage in creative, imaginative writing. Imagination, or fantasy, relies on stories and retrievals of one’s previous experiences that are combined and reworked based on certain elements of these past experiences in a creative manner to generate new propositions and scenarios [16].

We consider that creativity in writing imaginative narratives is important for psychological distress detection. A meta-analysis on trait creativity and psychopathology showed that mild psychopathology is associated with increased creativity, to the extent that it does not impair day-to-day functioning [17]. Similarly, external ratings of creativity in visual art were found to be associated with dehydroepiandrosterone-sulfate (DHEA-S), an endocrine marker of depression. Specifically, lower levels of DHEA-S (indicating an increased risk of depression) were predictive of higher creativity ratings, and this relationship was significantly moderated by the individual’s emotional vulnerability to social rejection or acceptance [18].

Accordingly, individuals’ mental health states should be reflected in the creativity of their narrative content, and analysis of these narratives may function as effective early detectors of psychological distress. Furthermore, imaginative free-text writing tasks may be more suited for mental illness detection in children and adolescents. As creativity refers to the ability to imagine new and useful things or situations [19], it involves imagining something that does not presently exist in reality. Thus, we propose that by constraining free-text narrative tasks to imaginative writing, we can effectively detect adolescent writers with possible psychopathological tendencies through examining the creativity of the written narratives as quantified by the amount of variation in their vocabulary and language use.

### Objectives

Based on the hypothesis outlined above, our research focuses on the detection of psychological distress in written imaginative narratives. To quantify these variations in vocabulary and language use, we applied NLP tools such as stylometric measures and lexicon-based sentiment analysis to analyze these texts. Although the act of analyzing stories itself is not a new concept, the use of NLP tools to quantify qualitative text in analyzing imaginative narratives remains a challenging approach. Nevertheless, we propose that written stories from youth at high and low risk of psychological distress can be differentiated through stylometric text analysis methods.

## Methods

### Participants

A total of 634 participants were initially recruited from Yahoo! Crowdsourcing, a Japanese online crowdsourcing platform. Participants were reimbursed 5 yen (~US $0.05) through Yahoo! Crowdsourcing. After excluding participants with meaningless words and reprints of copyrighted works in their responses, a total of 629 (267 males, 335 females, 27 no disclosed gender; mean age 40.9 years, SD 12.3) participants were identified. Note that age was approximated from the mean of the class (group) data, since we only collected information on the age group. Of these participants, we further narrowed our focus to responses from young participants. Following the World Health Organization definition of “youth” as individuals aged between 15 and 25 years, we examined a final 52 participants (14 males, 29 females, 9 did not disclose gender; mean age 19.8 years, SD 3.1). Although the main focus of the analysis was on youth, we also examined supplementary data on 577 adults (253 males, 306 females, 18 did not disclose gender; mean age 42.4 years, SD 12.3) to allow for the possibility of identifying youth-specific characteristics. During the first 2 weeks, we limited recruitment to users under the age of 25 years, but had low participation rates. Therefore, for the next 2 weeks, we recruited users without any age restriction and then selected only users of the target age group. As a result, we collected data from the 52 youth and 577 adults. The adult data were not intended to be part of our research, but showed sufficient potential to provide a developmental perspective, and therefore were included for use in the supplemental analysis.

### Materials

Participants were first instructed to “write an introduction to a story, with at least 200 (Japanese) characters.” This was an open-ended response task, designed to let participants engage in narrative writing by creating the introduction to an imagined story. Subsequently, they were assessed for psychological distress using the Kessler Psychological Distress Scale (K-10), a brief 10-item test widely used in screening mental illness (eg, in the World Health Organization World Mental Health Survey [20]). The cutoff value for K-10 was set at 30, which is a reference value to indicate a respondent’s state of severe psychological distress. Participants with a K-10 score ≥30 were assigned to the higher distress group and the remaining participants were assigned to the lower distress group.

### NLP Measures

We adopted stylometric measures for authorship detection that have been shown to have relationships with the attitudes and psychological tendencies of authors. We utilized 12 types of stylometrics, as listed in Table 1, based on Japanese text metrics organized in Asaishi [21]. Additionally, we performed sentiment analyses to examine the ratio of specific emotion terms (eg, happiness, surprise, anger; see Table 1) to the total number of terms in the text.

To reduce confounds related to story length, we limited the open-ended responses to include only the first 200 characters while preserving the sentence unit.

That is, we kept the maximum number of the introductory sentences if the last sentence (a) did not exceed the 200-character limit or (b) exceeded the limit by fewer characters than the characters from the end of its previous sentence. During this process, sentences were separated by punctuation marks, except for those inside parentheses.

Because some metrics require grammatical information, we applied shallow natural language parsing techniques. Tokenization and part-of-speech tagging were processed through the morphological analyzer MeCab [22].

Note that Japanese text is not tokenized by authors with spaces. CaboCha [23] was used for syntax parsing.

**Table 1:** Stylometric measures (value format).

Stylometric	Description (value format)
Percentages of character types	The ratios (%) of hiragana, katakana, and kanji (Chinese characters) to the characters in the story
Type token ratio	The ratio (%) of different words to the total number of words in the story
Percentages of content words	The ratio (%) of content words (ie, nouns, verbs, adjectives, and adverbs) to the total number of words in the story
Modifying words and verb ratio	The ratio (%) of verbs to adjectives, adverbs, and conjunctions for the words in the story; this stylometric has been used as one of the indicators of author estimation [24]
Percentages of proper nouns	The ratio (%) of proper nouns (named entities) to all words in the story
Word abstraction	The abstraction degrees of the words in the story. The abstraction degrees were obtained from the Japanese word-abstraction dictionary AWD-J [25] (real number)
Ratios of emotional words	The ratios (%), relative to all words in the story, of words associated with each of the following seven categories of emotions: sadness, anxiety, anger, disgust, trust, surprise, and happiness. Weights are assigned such that each value spans between 0 and 1; the sum of all values is 1. The degree of association with emotion was determined according to the Japanese emotional-word dictionary JIWC [26]
Number of sentences	The total number of sentences that make up the story (integer)
Length of sentences	Descriptive statistics for the number of characters in each sentence that constitutes the story. In particular, the average sentence length has been suggested to be linked to the writer’s creative attitude and personality [27] (real number)
Percentage of conversational sentences	Percentage of the total number of conversational sentences contained in the story
Depth of syntax tree	Descriptive statistics calculated for the depth of the dependency tree for each sentence in the story (real number)
Mean of the number of chunks per sentence	Descriptive statistics calculated for the average values of the number of chunks for each sentence in the story (real number)
Mean of the words per chunk	Descriptive statistics calculated for the average values of the number of words per chunk in the story (real number)

## Results

The results of the comparison of language indicators between youth and adults are shown in Table 2. See Multimedia Appendix 1 for examples of narrative writing in youth and adults.

In contrast to our hypothesis, participants in the higher distress group did not show significant increases in word richness or diversity. Most of the stylometrics that examined variation in word use (eg, type token ratio) did not significantly differ between groups. In exploring additional differences in language content between the higher and lower distress groups for youth, significant differences were observed in emotion terms for happiness-related word ratios. This suggests that narratives written by participants in the higher distress group were more likely to use happiness-related phrases and words than those in the lower distress group. No other significant differences were observed.

Although outside the scope of this study, we also carried out a supplementary analysis of narratives written by adults using the same procedure. In the adult participants, there were significant differences between higher and lower distress groups in content words, sadness, and the mean number of words per sentence clause. The higher distress group used fewer content words, fewer emotion words about sadness, and fewer words per chunk than the lower distress group (Table 2).

**Table 2:** Comparison of mean values of stylometric measures.

Stylometric		Youth					Adults			
		Higher distress (n=21), mean (SD)	Lower distress (n=31), mean (SD)	*t*^a^ (*df*=50)	*P* value	Higher distress (n=94), mean (SD)		Lower distress (n=483), mean (SD)	*t*^a^ (*df*=575)	*P* value
**Character types**										
	Hiragana	0.597 (0.079)	0.610 (0.075)	–0.591	.56	0.604 (0.087)		0.596 (0.084)	0.787	.43
	Katakana	0.060 (0.058)	0.047 (0.036)	0.990	.33	0.0463 (0.053)		0.597 (0.084)	0.491	.62
	Kanji (Chinese characters)	0.255 (0.053)	0.250 (0.062)	0.289	.77	0.268 (0.064)		0.276 (0.069)	–1.045	.30
Type token ratio		0.540 (0.075)	0.528 (0.058)	0.628	.53	0.550 (0.056)		0.542 (0.058)	1.168	.24
Content words		0.272 (0.068)	0.265 (0.053)	0.383	.70	2.490 (0.214)		2.548 (0.219)	–2.175	.03
Modifying words and verb ratio		0.394 (0.208)	0.424 (0.185)	–0.541	.59	0.406 (0.230)		0.393 (0.219)	0.504	.61
Proper nouns		0.010 (0.025)	0.009 (0.021)	0.152	.88	0.007 (0.018)		0.007 (0.013)	-0.056	.96
**Word abstraction**										
	Maximum	3.133 (0.184)	3.071 (0.145)	1.364	.18	3.057 (0.181)		3.060 (0.183)	–0.153	.88
	Average of the top 5 words	2.909 (0.105)	2.899 (0.091)	0.360	.72	2.878 (0.104)		2.886 (0.105)	0.509	.66
**Emotional words**										
	Sadness	0.103 (0.010)	0.107 (0.012)	–1.223	.23	0.104 (0.016)		0.108 (0.020)	–2.051	.04
	Anxiety	0.105 (0.017)	0.109 (0.023)	–0.690	.50	0.105 (0.024)		0.108 (0.020)	–1.402	.16
	Anger	0.172 (0.021)	0.177 (0.018)	–0.956	.34	0.174 (0.024)		0.171 (0.021)	1.372	.17
	Disgust	0.167 (0.036)	0.174 (0.039)	–0.731	.47	0.172 (0.041)		0.167 (0.034)	1.399	.16
	Trust	0.165 (0.014)	0.160 (0.017)	1.071	.29	0.165 (0.026)		0.162 (0.022)	1.133	.26
	Surprise	0.154 (0.019)	0.163 (0.015)	–1.967	.06	0.164 (0.028)		0.161 (0.022)	0.819	.41
	Happiness	0.134 (0.049)	0.109 (0.018)	–2.657	.01	0.116 (0.041)		0.123 (0.039)	–1.384	.17
Number of sentences		6.619 (2.578)	7.419 (2.566)	–1.101	.28	7.011 (2.975)		6.778 (2.454)	0.809	.42
Length of sentences		33.740 (10.807)	29.544 (11.924)	1.292	.20	30.761 (10.898)		33.962 (22.711)	–1.336	.18
Conversational sentences		0.045 (0.082)	0.062 (0.107)	–0.606	.55	0.034 (0.121)		0.042 (0.114)	–0.298	.77
The number of chunks per sentence		8.194 (2.872)	8.167 (3.704)	0.028	.98	7.756 (3.400)		8.033 (4.302)	–0.546	.59
The words per chunk		2.586 (0.305)	2.561 (0.270)	0.305	.76	2.490 (0.214)		2.548 (0.219)	–2.175	.03

^a^Since equal variances were not assumed, Welch *t*-tests were used to examine between-group differences on the above measures.

## Discussion

### Narrative Language Features and Youth Mental Health

We found significant differences between the lower and higher distress groups, particularly with regard to the relative frequency of happiness-related terms. Happiness is commonly viewed as a positively valenced emotion across cultures [28]. At a glance, the increased usage of happiness-related words in the higher distress group suggests that youth with increased tendencies to psychological distress or illness may prefer writing more positive narratives. Considering that the concept of happiness is not typically associated with psychological distress, our result suggesting the stronger presence of happiness-related terms in the higher psychological distress group appears contradictory. Nevertheless, we posit two potential explanations for this result. First, these findings may reflect the possibility that youth with higher distress prefer happier stories. Alternatively, these findings may suggest that more frequent priming of happiness may induce psychological distress in youth. Although a causal relationship cannot be investigated from this single cross-sectional study, we speculate on several interpretations. Prior research on self-directed narratives has revealed an association with depression. For example, those who tended to imagine a positively inclined future for themselves had lower depression measures (eg, Center for Epidemiological Studies Depression Scale, Children’s Depression Inventory, Beck Depression Inventory) at the time of their imagination, but in subsequent follow-up, they were found to have higher tendencies for depression than the group that had anticipated a less positive future [29].

By disambiguating these two concepts, one possibility could be that participants did not need to consider their “self” (current circumstances) in creative writing, and were able to imagine freely without these constraints. Thus, the increased positive affect (happiness scores) in their writing may suggest that this was more idealized or desired. Such an interpretation would be consistent with past research linking an increased desire for happiness with depression [30].

However, we reiterate that these explanations are speculations based on the pattern of results obtained from our data. More research is needed to confirm the applicability of these explanations in creative writing for individuals with tendencies toward psychological distress.

### Type Token Ratio and Word Abstraction

Our results showed no significant difference in vocabulary usage, measured in this study through indicators such as the type token ratio, nor in the abstraction of the words for the narrative passages. One explanation could be that the memories of one’s own experiences do not necessarily affect the generation of a story. Differentiation according to the level of word abstraction alone is insufficient, because narratives may be conceptualized separately from one’s own experiences, referencing the collective knowledge from cultural media and the experiences of others.

### Pros and Cons of Crowdsourcing

Several previous studies have shown that crowdsourcing is an appropriate tool for recruiting research participants [31,32]. Nevertheless, we acknowledge some problems with crowdsourcing data collection. For example, participants may attempt the same questionnaire twice, and incorrect comprehension of the instructional text may be problematic due to participation from nonnative speakers. Another problem we had to face was the phenomenon of “satisficing,” in that participants may tend to conserve cognitive resources in survey research [33]. In crowdsourcing, people are often motivated to work on multiple tasks in as short a time as possible to increase their monetary reward because of low unit costs.

In this study, we took steps to reduce these problematic effects from crowdsourcing by using a service in which native speakers (in this case, Japanese speakers) form the vast majority of users. We also excluded duplicated IDs and data (narratives) that were directly lifted from copyrighted works or lists of meaningless words. Although we cannot definitively rule out any bias or duplicate participants in our research, precautions were taken to safeguard against these potential problems.

### Limitations and Future Directions

We note several limitations of our study. First, we had limitations with regard to the age of our sample. The crowdsourcing platform had a minimum age of 15, meaning that adolescents under 15 years old were not included in this study. Second, we used the K-10 for screening purposes, which is a general questionnaire and lacks sensitivity to specific diagnoses. Thus, there is a possibility that different types of psychopathological tendencies may exhibit differentiated effects or underlying mechanisms. Future studies should consider the use of actual clinical diagnoses as the criteria for mental illness beyond crowdsourced convenience sampling.

Finally, the linguistic indicators used in this study are widely used for author estimation and are usually applied to large volumes of text. In this study, narrative sentences of about 200 characters each were used, which may be insufficient for detecting the presence of true effects. Consequently, we are unable to rule out the use of creativity measures in imaginative writing as a means for detecting psychological distress, as our lack of a significant finding in this aspect can also be explained by an insufficiency in the length of our narrative data.

Nevertheless, our study lays the groundwork for psychological distress surveillance programs in sensitive populations, especially in situations where recollection of personal, self-related narratives or self-disclosure may be problematic or risky. This may be more relevant in the Japanese context. Japanese undergraduate students were less likely to self-disclose experiences of bullying compared to a US sample, and this was fueled by a concern for disrupting social and relational harmony [34]. As such, surveillance methods that rely on fictional narrative content without reliance on self-disclosure may be more suitable in a collectivistic Japanese context. Our exploratory result identifies happiness and surprise-related content as potential indicators of psychological distress in the writer. Future research should confirm these findings on a larger scale, with a larger, preregistered study involving more diverse samples and cross-cultural comparisons. Narrative writing may even provide therapeutic benefits for individuals suffering from depression [35], and future studies can also quantify the effectiveness of such methods in randomized controlled trials.

### Conclusions

Youth with tendencies toward mental illness were found to write more positive stories that contained more happiness-related terms. Although the mechanisms underlying these differences in frequency of happiness-related term usage are speculative at present, these results may potentially have more widespread implications on screening, particularly in cultures such as Japan that are not accustomed to self-disclosure. This is a preliminary finding and more confirmatory research is needed to establish the robustness of these results.

## Appendix

Multimedia Appendix 1 Examples of narrative writing. Shows the highest/lowest type token ratio and ratio of happiness-related words for youth and adults.

## Abbreviations

DHEA-S: dehydroepiandrosterone-sulfate
K-10: Kessler Psychological Distress Scale
NLP: natural language processing

## References

[1] Kessler RC, Amminger GP, Aguilar-Gaxiola S, Alonso J, Lee S, Ustün TB (2007). Age of onset of mental disorders: a review of recent literature. Curr Opin Psychiatry.

[2] Stallman HM (2010). Psychological distress in university students: A comparison with general population data. Austral Psychol.

[3] Moylan S, Maes M, Wray NR, Berk M (2013). The neuroprogressive nature of major depressive disorder: pathways to disease evolution and resistance, and therapeutic implications. Mol Psychiatry.

[4] Rice F, Riglin L, Lomax T, Souter E, Potter R, Smith D, Thapar A, Thapar A (2019). Adolescent and adult differences in major depression symptom profiles. J Affect Disord.

[5] Wight RG, Sepúlveda JE, Aneshensel CS (2004). Depressive symptoms: how do adolescents compare with adults?. J Adolesc Health.

[6] Jin H, Wu S (2020). Text messaging as a screening tool for depression and related conditions in underserved, predominantly minority safety net primary care patients: validity study. J Med Internet Res.

[7] Topaz M, Adams V, Wilson P, Woo K, Ryvicker M (2020). Free-text documentation of dementia symptoms in home healthcare: a natural language processing study. Gerontol Geriatr Med.

[8] Morales M, Scherer S, Levitan R (2017). Cross-modal review of indicators for depression detection systems.

[9] Liu Y, Yu X, Yang B, Zhang F, Zou W, Na A, Zhao X, Yin G (2017). Rumination mediates the relationship between overgeneral autobiographical memory and depression in patients with major depressive disorder. BMC Psychiatry.

[10] Matsumoto N, Mochizuki S (2013). Reduced autobiographical memory specificity due to depression: Assess the instructions for analog study. Japanese J Res Emotions.

[11] Williams JMG, Teasdale JD, Segal ZV, Soulsby J (2000). Mindfulness-based cognitive therapy reduces overgeneral autobiographical memory in formerly depressed patients. J Abnorm Psychol.

[12] Kuyken W, Howell R, Dalgleish T (2006). Overgeneral autobiographical memory in depressed adolescents with, versus without, a reported history of trauma. J Abnorm Psychol.

[13] Asai A, Barnlund D (1998). Boundaries of the unconscious, private, and public self in Japanese and Americans: a cross-cultural comparison. Int J Intercult Relation.

[14] Schug J, Yuki M, Maddux W (2010). Relational mobility explains between- and within-culture differences in self-disclosure to close friends. Psychol Sci.

[15] Best P, Manktelow R, Taylor B (2014). Online communication, social media and adolescent wellbeing: A systematic narrative review. Child Youth Serv Rev.

[16] Vygotsky LS (2014). Imagination and creativity in childhood. J Russian East Eur Psychol.

[17] Paek SH, Abdulla AM, Cramond B (2016). A meta-analysis of the relationship between three common psychopathologies—ADHD, anxiety, and depression—and indicators of Little-c Creativity. Gift Child Quart.

[18] Akinola M, Mendes WB (2008). The dark side of creativity: biological vulnerability and negative emotions lead to greater artistic creativity. Pers Soc Psychol Bull.

[19] Stein MI (1953). Creativity and culture. J Psychol.

[20] Kessler RC, Andrews G, Colpe LJ, Hiripi E, Mroczek DK, Normand SLT, Walters EE, Zaslavsky AM (2002). Short screening scales to monitor population prevalences and trends in non-specific psychological distress. Psychol Med.

[21] Takuma A (2017). An overview of indicators for quantifying text features [in Japanese]. J Japan Soc Library Inf Sci.

[22] MeCab: Yet Another Part-of-Speech and Morphological Analyzer.

[23] CaboCha: Yet Another Japanese Dependency Structure Analyze.

[24] Watanabe M (2003). Statistical analysis of the difference between original novels written by Haruki Murakami and foreign novels translated. Math Ling.

[25] AWD-J: Abstractness of Word Database for Japanese common words.

[26] JIWC-Dictionary. GitHub.

[27] Takeshige S, Inoue N (1986). The writing style of Yasushi Inoue in his short novels. Dokusho Kagaku (The Science of Reading).

[28] An S, Ji L, Marks M, Zhang Z (2017). Corrigendum: Two sides of emotion: exploring positivity and negativity in six basic emotions across cultures. Front Psychol.

[29] Oettingen G, Mayer D, Portnow S (2016). Pleasure now, pain later: positive fantasies about the future predict symptoms of depression. Psychol Sci.

[30] Mahmoodi Kahriz B, Bower J, Glover F, Vogt J (2019). Wanting to be happy but not knowing how: poor attentional control and emotion-regulation abilities mediate the association between valuing happiness and depression. J Happiness Stud.

[31] Clifford S, Jewell RM, Waggoner PD (2015). Are samples drawn from Mechanical Turk valid for research on political ideology?. Res Politics.

[32] Majima Y (2017). The feasibility of a Japanese crowdsourcing service for experimental research in psychology. SAGE Open.

[33] Holland JL, Christian LM (2008). The influence of topic interest and interactive probing on responses to open-ended questions in web surveys. Soc Sci Comput Rev.

[34] Matsunaga M (2010). Individual dispositions and interpersonal concerns underlying bullied victims’ self-disclosure in Japan and the US. J Soc Person Relation.

[35] Cooper P (2013). Writing for depression in health care. Br J Occup Ther.

